# Vestibular rehabilitation therapy in combination with transcranial direct current stimulation (tDCS) for treatment of chronic vestibular dysfunction in the elderly: a double-blind randomized controlled trial

**DOI:** 10.1016/j.bjorl.2020.11.004

**Published:** 2020-11-29

**Authors:** Nader Saki, Arash Bayat, Soheila Nikakhlagh, Golshan Mirmomeni

**Affiliations:** a Ahvaz Jundishapur University of Medical Sciences, Hearing Research Center, Ahvaz, Iran; b Ahvaz Jundishapur University of Medical Sciences, School of Rehabilitation Sciences, Musculoskeletal Rehabilitation Center, Ahvaz, Iran; c Ahvaz Jundishapur University of Medical Sciences, Students Research Committee, Ahvaz, Iran

**Keywords:** Transcranial direct current stimulation, Vestibular rehabilitation, Elderly, Anxiety, Balance

## Abstract

**Introduction:**

Dizziness and imbalance are common dysfunctions in the elderly. Vestibular rehabilitation therapy is an effective method to alleviate chronic dizziness in patients with vestibular dysfunction. Transcranial direct current stimulation has reportedly improved balance function in patients with vestibular dysfunction.

**Objective:**

This study was conducted to investigate the therapeutic efficacy of vestibular rehabilitation combined with transcranial direct current stimulation in elderly patients with vestibular dysfunction.

**Methods:**

In a double-blinded randomized controlled trial, 36 elderly patients with chronic vestibular dysfunction were randomly assigned to either vestibular rehabilitation and transcranial direct current stimulation (n = 18) or vestibular rehabilitation alone (n = 18) group. The transcranial stimulation protocol consisted of multisession bifrontal electrical stimulation of the dorsolateral prefrontal cortex (2 mA intensity and 20 min duration), followed by rehabilitation exercises. The vestibular rehabilitation protocol consisted of habituation and adaptation exercises combined with gait exercises during a three week period. The primary outcome of this study was the dizziness handicap inventory score, and the secondary outcomes were activities-specific balance confidence and Beck anxiety inventory scores.

**Results:**

For the dizziness handicap score, the repeated-measures analysis of variance showed a significant main effect of “time”, “stimulation” and stimulation × time interaction effect. There was a significant reduction in the overall dizziness handicap score with “time” for both the groups, which was more pronounced in the vestibular rehabilitation and electrical stimulation group. In terms of activities-specific balance confidence change scores, we found a significant main effect of “time” and “stimulation” main factors, but this effect for stimulation × time interaction was not significant. For the Beck anxiety score, we observed a significant main effect of “time”, but no evidence for the main effect of the “stimulation” factor.

**Conclusion:**

Bifrontal transcranial direct current stimulation in combination with vestibular rehabilitation therapy is a promising approach to improve chronic vestibular symptoms in the elderly.

## Introduction

Dizziness and imbalance are common complaints among older people. It has been estimated that about 30% of people older than 65 years’ experience some forms of dizziness, increasing to 50% in people older than 80 years.^1^ Dizziness in the elderly is a growing public health concern because older people who suffer from dizziness have a significantly higher risk of accidental falls and consequent injuries.^2^, ^3^, ^4^ The underlying cause of dizziness in the elderly patients is very complicated because there are many contributing mechanisms.

Vestibular Rehabilitation Therapy (VRT) is an exercise-based therapeutic program to improve balance function in patients with significant deficits of vestibular origin.^5^, ^6^ VRT exercises aim to reduce the disabling symptoms through the central mechanisms of neuroplasticity, including habituation, adaptation and substitution, which accelerate the vestibular compensation process.^7^, ^8^, ^9^

Recently, modulation of cortical and sub-cortical excitability through non-invasive methods such as non-invasive brain stimulation has received increasing attention as a means to improve performance during the course of therapy. The transcranial Direct Current Stimulation (tDCS) is a safe and non-invasive neuromodulation technique that can modulate neural activities. It applies a weak direct current to the brain through anodal and cathodal electrodes placed on the scalp, which can modulate cortical excitability and result in physiological and behavioral alternations to improve functional performance.^10^, ^11^ It has been shown that anodal stimulation has an excitatory effect on the underlying cerebral cortex by depolarizing neurons, while cathodal stimulation induces hyperpolarization of the underlying neurons, mainly by affecting the resting membrane potential.^12^, ^13^ Recent evidences have shown the benefit of tDCS on motor learning, postural control, gait, and working memory.^14^, ^15^, ^16^, ^17^, ^18^, ^19^

The present study aims to comparatively investigate the therapeutic effects of VRT alone and in combination with tDCS on vestibular dysfunctions in elderly patients. The main hypothesis is that combined VRT-tDCS could lead to a higher therapeutic effect than the VRT alone. The main hypothesis is that a combined VRT-tDCS protocol results in a greater improvement in balance performance compared with the VRT alone approach in elderly patients with vestibular dysfunction. The underlying assumption is that the tDCS would prime the central nervous system and thus create a stronger and faster learning effect for adapting to vestibular deficits.

## Methods

### Participants

Thirty-six elderly patients with chronic vestibular dysfunction who were treated with a customized vestibular rehabilitation program participated in this study. All patients suffered from medication-resistant and chronic (more than 2-year duration) vertigo. The patients underwent at least two weeks of medication washout period before the first therapy session. The diagnosis of vestibular dysfunction was based on the detailed medical case history, video Head Impulse Test (vHIT), Videonystagmography (VNG) including positioning/positional, bithermal caloric, and oculomotor tests, and cervical Vestibular Evoked Myogenic Potentials (cVEMP) tests. The inclusion criteria included age range of 65–80 years, diagnosis of chronic vestibular dysfunction, and normal or corrected-to-normal vision with an optional aid. Individuals with a history of acute and recurrent vestibular dysfunction, use of medicines with potential vestibular adverse effects, brain trauma, metallic implants in head or neck near to the site of stimulation, orthopedic limitations interfering with the study, presence of any psychiatric disorders, and epileptic seizures were excluded.

All the experimental procedures of this study were approved by the local Research Ethics Board (Registration number: IR.AJUMS.REC.1399.327), which were in accordance with the ethical standards of the Helsinki declaration (1964). After the enrolment and before the start of the experimental procedures, the researchers clearly explained the objectives, possible benefits, and side effects of the study to the participants. All patients signed a written informed consent before participating in the study.

### Study protocol

**Figure 1:**
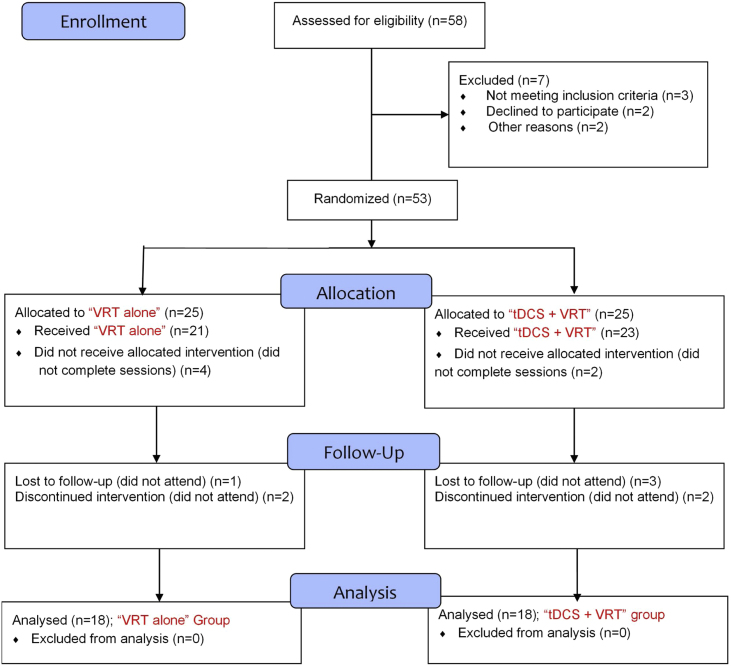
Consort diagram of the study. VRT, Vestibular Rehabilitation Therapy; tDCS, transcranial Direct Current Stimulation.

In a double-blind, randomized controlled design, eligible patients were randomly assigned to either “VRT-tDCS” (combined VRT and tDCS) or “VRT alone” treatment by the study coordinators. Both groups were matched for age, gender, and disease duration (Table 1). The CONSORT flow diagram of the study is presented in Fig. 1. To reduce the procedure and subjective bias, the patients and the researchers were blinded to the type of protocol. Only the clinician conducting the tDCS procedure and the physician assigning the randomization were aware of the group information.

**Table 1:** Comparison of demographic characteristics of participants between VRT-tDCS (n = 18) and VRT alone (n = 18) groups.

Variable	Group		p-value	Test
	VRT	VRT-tDCS		
Age (years)	71.33 ± 6.16^a^	72.11 ± 5.09^a^	*t* = 0.45; *p* = 0.65	Independent sample *t*
Dizziness duration (years)	3.94 ± 1.21^a^	4.50 ± 2.03^a^	*t* = 0.99; *p* = 0.32	Independent sample *t*
Gender	11 M; 7 F	10 M; 8 F	X^2^ = 25.9; *p* = 0.735	Chi-square

VRT, Vestibular Rehabilitation Therapy.

a Values are in mean ± SD or n (%).

### Vestibular rehabilitation therapy

The vestibular exercises were administered to each patient according to the patient’s complaint and perceived functional limitations established in the history-taking examinations. All patients were treated with a 3 week course of a staged vestibular rehabilitation plan. During the first two weeks, the patient attended a series of 25–30 minute exercise sessions, 6 days a week (total of 12 sessions). Then, the patient continued to conduct the VRT exercises at home, with a written home exercise plan and instructions, on a daily basis, for the last week.

The therapy program consisted of “habituation” and “adaptation” exercises in combination with gait exercises. During the “habituation” training course, the head was moved to the left and right side, while the eyes were kept fixed on a specific target. In order to perform “adaptation” exercises, the movements that provoke the patient’s symptoms were identified and the patient conducted these exercises until they no longer responded adversely to the stimuli. “Gait” exercises were also performed to improve postural stability through the enhancement of static and dynamic postures. In the current study, walking began on flat surfaces, and then proceeded to uneven surfaces. The task difficulty was subsequently increased by adding head rotations in the form of right and left headshaking while walking on a hard surface.

### Transcranial direct current stimulation

**Figure 2:**
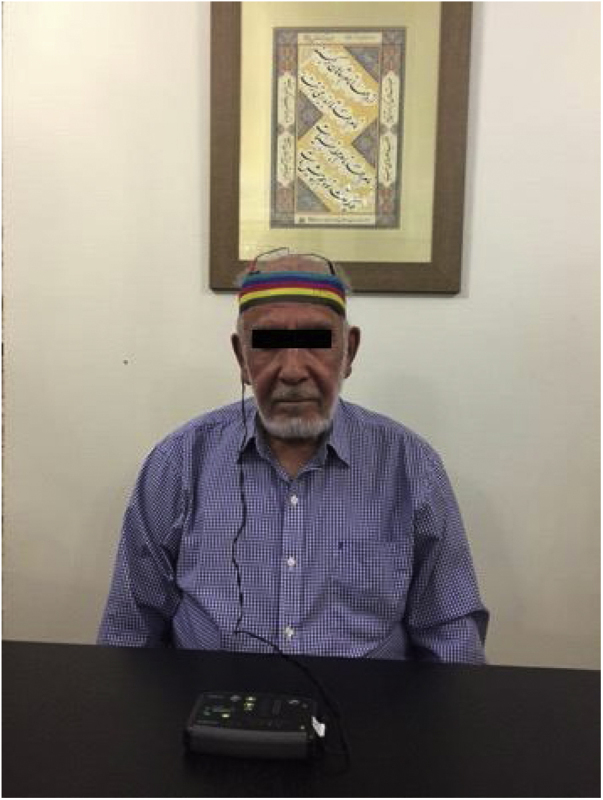
tDCS electrode configuration.

The tDCS was applied via two electrodes embedded in a pair of saline-soaked sponges (35 cm^2^) and delivered through a battery-driven DC stimulator (OASIS Pro™ system, Canada). The 10–20 electroencephalogram electrode positioning system was utilized for determining the site of electrodes. The anode and cathode electrodes were placed over the right (F4) and left DLPFC (F3) sites and during the stimulation the impedance of the electrodes was consistently kept below 3 kΩ (Fig. 2). In each tDCS session, 2 mA current was delivered for 20 min. Stimulation was applied on 6 consecutive days during a 3 week period (18 sessions in total).

### Clinical evaluation

The primary outcome was alternation in the impact of dizziness on daily function and quality of life that was measured using the Persian version of the Dizziness Handicap Inventory (DHI). The DHI consists of 25 self-assessment questions to measure the physical (7 items), emotional (9 items), and functional (9 items) domains of disability related to vestibular disturbances. Each item provides a choice of 3 responses: “no” (0 points), “sometimes” (2 points), or “yes” (4 points). The DHI scores range from 0 to 100, where 0 corresponds “no handicap” and 100 corresponds to “greatest” self-perceived handicap. The symptoms were categorized as mild, moderate, or severe if the DHI scores range 0–30, 31–60, and 61–100, respectively.

Secondary subjective outcomes included the Activities-specific Balance Confidence (ABC) scale. The ABC is a self-reported questionnaire providing information on balance confidence in the performance of various daily activities without loss of balance and fear of falling. The ABC consists of 16 items scored on a range from 0% to 100% confidence (0 indicating no confidence and 100 indicating full confidence).

The Beck Anxiety Inventory (BAI) was also utilized to assess anxiety-related to vestibular disturbances at pre- and post-intervention phases. The BAI consists of 21 items rated on a 4-point Likert scale from 0 (not at all) to 3 (severely). Then, the total score has a minimum of 0 and a maximum of 63. A lower score of BAI indicates a lower level of dizziness related anxiety.

The DHI, BAI, and ABC scales were assessed before the first VRT session, after the 1-week, 2 weeks and 3 weeks of followup in both groups.

### Statistical analysis

The primary outcome of this study was the DHI score and secondary outcome measures were ABC and BAI scores. For nominal data (e.g., gender), chi-square tests were used to compare VRT-tDCS and “VRT alone” groups. Independent sample *t*-tests were also used to determine if individual baseline measures (DHI, BAI and ABC) differed between groups. Prior to conducting *t*-tests, Levene’s test for equality of variances was used. Levene’s test was not violated in any case indicating the assumption of the equality of variances. Mixed RM-ANOVAs with the within‐subject factor Time and the between‐subject factor Stimulation were performed for evaluating changes in DHI, ABC, and (DHI, BAI, and ABC). For the ANOVAs, sphericity was tested with the Mauchly's test, and in the event of a violation of Mauchly’s test, the Greenhouse-Geisser correction was applied. In case of significant effects, followup post-hoc *t*-tests were carried out using LSD adjustments for multiple comparisons to examine if tDCS caused a significant difference relative to “VRT alone” or baseline. All statistical tests were performed using SPSS 25 (SPSS Inc., Chicago, IL, USA); *p*-value < 0.05 was set a statistically significant for all inferential statistics.

## Results

The average age of the participants was 71.17-years, and the female/male ratio was 15:21. Of the 36 patients, 17 patients with Benign Paroxysmal Positional Vertigo (BPPV), 13 with Meniere’s disease, and 6 with vestibular migraine were diagnosed.

The demographic information and baseline clinical characteristics of the participants did not differ significantly between the VRT-tDCS and “VRT alone” groups (Table 2). Chi-square test showed a similar proportion of males and females across two groups (X^2^ = 0.78, *p* =  0.55), and that the duration of dizziness was proportionate across groups (*p* =  0.56). Furthermore, the baseline primary and secondary outcome measures did not differ significantly between both groups (Table 3).

**Table 2:** Comparison of baseline primary and secondary outcome measures between VRT-tDCS (n = 18) and VRT alone (n = 18) groups.

Variable	VRT a	VRT-tDCS a	p-value	Test
DHI	45.44 ± 8.140	44.01 ± 7.577	*t* = -0.55; *p* = 0.585	Independent Sample *t*
BAI	24.89 ± 2.518	25.28 ± 3.140	*t* = 0.41; *p* = 0.389	
ABC	54.44 ± 5.596	53.78 ± 6.208	*t* = -0.33; *p* = 0.073	

VRT, Vestibular Rehabilitation Therapy; ABC, Activities-specific Balance Confidence; BAI, Beck Anxiety Inventory; DHI, Dizziness Handicap Inventory.

a Values are in mean ± SD.

**Table 3:** Results on self-report inventories at baseline and final therapy sessions for VRT-tDCS (n = 18) and VRT alone (n = 18) groups.

Measure	Pre-treatment a	Post-treatment a	*p*-value
DHI			
VRT	45.44 ± 8.14	23.01 ± 9.84	< 0.001
VRT-tDCS	44.01 ± 7.57	18.22 ± 4.16	< 0.001
BAI			
VRT	24.89 ± 2.51	16.56 ± 3.82	< 0.001
VRT-tDCS	25.28 ± 3.14	14.39 ± 4.23	0.008
ABC			
VRT	54.44 ± 5.59	71.89 ± 4.31	< 0.001
VRT-tDCS	53.78 ± 6.21	79.11 ± 7.42	< 0.001

VRT, Vestibular Rehabilitation Therapy; DHI, Dizziness Handicap Inventory; ABC, Activities-specific Balance Confidence; BAI, Beck Anxiety Inventory.

a Values are in mean ± SD.

For the primary outcome parameter, DHI, the RM-ANOVA showed a significant main effect of “Time” (F = 114.179, *p* <  0.001) and “Stimulation” (F = 5.012, *p* <  0.032), but not for Stimulation × Time interaction effect (F = 0.549, *p* =  0.651). This analysis revealed no significant effect for “disease duration” (*p* =  0.034), or Time × “disease duration” (*p* =  0.245) interaction effect. Post-hoc comparisons of VRT-tDCS and VRT alone groups revealed that tDCS stimulation resulted in a significant difference in DHI scores across all time points between both groups. A consecutive one-way ANOVA revealed a significant effect of “Time” (*p* <  0.001) in the combined VRT-tDCS group.

In terms of ABC change scores, there was no significant difference in the ABC scores between VRT-tDCS and “VRT alone” group treatment prior to tDCS stimulation (Independent samples *t*-test, *p* =  0.891). The RM-ANOVA exhibited a significant main effect of “Time” (F = 1145.65, *p* <  0.001), “Stimulation” (F = 6.113, *p* =  0.019) and Time × Stimulation interaction (F = 5.181, *p* =  0.002). There was neither a significant main effect of “disease duration” (*p* =  0.083), nor disease duration × Time (*p* =  0.123).

For the BAI score, we observed a significant main effect of “Time” (F = 182.76, *p* <  0.001), but no evidence for Time × Stimulation interaction (F = 1.501, *p* =  0.219) and Stimulation (F = 1.790, *p* =  0.191) indicating no consistent difference between VRT-tDCS and “VRT alone” groups over all time points (Table 4).

**Table 4:** Results of the repeated-measures ANOVA conducted for THI, ABC, and BAI scales across different time intervals (each 4 time points).

Measure	Source	df	F	Sig.	$\eta^{2}$
DHI	Time	3	114.17	< 0.001	0.771
	Time * Stimulation	3	0.549	0.651	0.016
	Stimulation	1	5.012	0.032	0.128
ABC	Time	3	1145.65	< 0.001	0.811
	Time * Stimulation	3	5.181	0.002	0.132
	Stimulation	1	6.113	0.019	0.154
BAI	Time	3	182.76	< 0.001	0.843
	Time * Stimulation	3	1.501	0.219	0.042
	Stimulation	1	1.790	0.191	0.051

BAI, Beck Anxiety Inventory; ABC, Activities-specific Balance Confidence, DHI, Dizziness Handicap Inventory.

### tDCS adverse effects

We designed a customized form to assess the adverse effects of tDCS.^20^ Our findings showed that itching was the most often reported adverse effect in both VRT-tDCS 23 (79.3%) and “VRT alone” 8 (53.3%) groups followed by headache, and fatigue (Table 5). However, we did not find any significant difference in the frequency of various tDCS adverse effects between the two groups (Chi-square test, *p* >  0.05). None of the patients reported tingling, skin irritation, or pinching at the tDCS contact site.

**Table 5:** Number of patients experiencing specified tDCS adverse effects in “tDCS in combination with VRT”, and “VRT alone” groups.

Side effect	“VRT + tDCS” Group (n = 18)		“VRT alone” Group (n = 18)		*p*-value
	Mild intensity	Moderate intensity	Mild intensity	Moderate intensity	
Itching	5	2	3	2	0.687
Fatigue	2	1	1	1	0.750
Headache	2	2	2	1	0.526

VRT, Vestibular Rehabilitation Therapy; tDCS, transcranial Direct Current Stimulation.

## Discussion

This study comparatively investigated the efficacy of combined VRT-tDCS and VRT alone on improving balance functions and the symptoms of vestibular dysfunction in elderly patients. Balance function disorder in the elderly is a very serious public health issue from both a clinical and an economic viewpoint, where its prevalence reaches 30% in the elderly older than 60 years. People aged 65 years or older have the highest risk of falling. Recent evidence have shown that the falling in older people may not only result in serious injury or death, but also may lead to increased depression and anxiety, and reduced quality of life.^21^, ^22^ It has been demonstrated that the number of vestibular hair cells is reduced in older adults compared to younger adults. However, the reduction in sensory hair cells is not uniform throughout the peripheral vestibular system. The semicircular canals experience approximately a 40% decline in hair cells, whereas otoliths (saccule and utricle) lose approximately 25% of their hair cells with increasing age.

Moreover, utricular sensory cells are more susceptible to age-related degeneration than saccular hair cells.^23^ The size and number of vestibular nerve fibers also decrease with increasing age, beginning around age 40. Fewer vestibular sensory cells and neural pathways lead to an age-related decrement in vestibular afferent signals to the central vestibular nervous system. There is also an associated reduction in the number of cerebellar cells that contribute to the modulation of vestibular afferents.^23^, ^24^, ^25^

VRT has been recommended as a beneficial therapeutic option in treating the elderly with chronic decompensated vestibular deficits.^26^, ^27^, ^28^ Several studies have demonstrated that VRT could improve postural stability, self-confidence, quality of life as well as reducing symptoms of emotional distress, depression and anxiety.^29^, ^30^, ^31^ The results of the current study showed that adaptation and habituation exercises in combination with tDCS exercises rapidly improved the symptoms, dizziness-related disability, and balance confidence in elderly with a chronic vestibular disorder. It is postulated that adaptation exercises, consisting of repeated head and eye movements, could help the central vestibular nervous system through rearrangement of Vestibular-Ocular Reflex (VOR) networking. Habituation (compensatory) exercises promote the vestibular compensation process using repetitive movements or provoking stimuli.^32^

The primary objective of this study was to identify whether combined VRT-tDCS, in comparison with the VRT alone, results in greater improvement in the dizziness and balance function in elderly patients with chronic vestibular dysfunction. During the last decade, tDCS has been extensively used in different neurological and neurocognitive disorders with promising outcomes in several disorders including depression, tinnitus, Alzheimer’s disease, attention deficit hyperactivity disorder, stroke rehabilitation as well as for improving cognitive functions in healthy individuals.^33^, ^34^, ^35^ We employed the bifrontal tDCS (anode/cathode over right/left dorsolateral prefrontal cortex or DLPFC) to improve vestibular symptoms. Previous studies have demonstrated that bifrontal tDCS could reduce peripheral drive through modulating neural correlates of balance function at various levels of the central vestibular system probably through a top-down mechanism.^36^, ^37^

We observed that total DHI scores at post-treatment phases were significantly reduced compared to the baseline scores in both groups. This reduction was more pronounced in the “VRT and tDCS” group compared to the “VRT alone” group, suggesting that multi-session tDCS is a beneficial method for the management of vestibular dysfunctions in the elderly population. The positive effect of bifrontal tDCS on vestibular symptoms in the elderly could be attributed to increased excitability of the right prefrontal cortex and decreased excitability of the left prefrontal cortex considering the sites of the anodal and cathodal electrodes. DLPFC area is involved in attention, working memory, and cognitive function. Furthermore, DLPFC plays a key role in gating, postural stability and, motor planning.^38^, ^39^ It seems that tDCS stimulation over the DLPFC area may prime the central vestibular system during therapeutic intervention, providing the potential to enhance synaptic plasticity and alleviate the chronic vestibular symptoms.

In a similar study, Koganemaru et al.^17^ investigated the treatment outcomes of VRT combined with transcranial cerebellar Direct Current Stimulation (tcDCS) in patients (n = 16) with chronic dizziness due to vestibular dysfunction. However, contrary to our study they used tDCS over the cerebellum, which was partially combined with VRT in which the patients received VRT concurrently with either 20-min real tDCS (2 mA) or sham stimulation for 5 days. DHI scores in the tDCS group showed significant improvement over those in the sham group. Their findings also demonstrated that combined VRT-tDCS appears to be a promising therapeutic approach in improving chronic dizziness due to vestibular dysfunction.

Arshad et al.^40^ investigated the effects of left cathodal tDCS over the parietal cortex on the modulation of vestibular function to assess whether tDCS induced asymmetries in the parietal excitability would modulate vestibular function. They aimed to understand the role of interhemispheric parietal balance in vestibular processing. They reported that left cathodal tDCS over the parietal cortex leads to an asymmetrical modulation of VOR. VOR reflex is an important component for gaze stabilization during head perturbations and is created by a combination of vestibular and retinal velocity inputs. Despite the significant contribution of brainstem centers in the VOR, higher-order integration of visuo-vestibular inputs may be critical for the conscious perception of body position in space and potentially to regulate reflexes such as the VOR. The findings of this study showed that cathodal tDCS over the left parietal cortex could disrupt parietal balance through inhibiting the left hemisphere in right-handed subjects that resulted in an asymmetrical suppression of the VOR. This implies that the right hemisphere is dominant for vestibular cortical processing. Therefore, tDCS over appropriate brain regions can be exploited to modulate the functions of vestibular processing.

De Moura et al.^41^ conducted a systematic review and meta-analysis of thirty studies on the effectiveness of tDCS on postural control to identify its efficacy, the most beneficial target brain areas and the effect on different populations. Their findings showed that tDCS could significantly improve the balance control observed as a reduction in the Center Of Pressure (COP) displacement area. The tDCS effects were greatest in individuals with Cerebral Palsy (CP) and healthy young adults. The most common regions for stimulation were the primary motor cortex (M1), prefrontal cortex, and cerebellum. Analysis of the effects of tDCS over different brain areas showed that stimulation over M1 resulted in the most significant effect. However, for the cerebellum and prefrontal cortex the findings were divergent. They concluded that tDCS could improve balance control and the impacts are more evident in healthy and CP subjects. The therapeutic effects were significant when tDCS is applied over the primary motor cortex.^41^

Our findings also revealed an improvement in the balance confidence for daily activities (ABC scores) following VRT exercises over time. It has been indicated that tDCS could regulate the premotor activation of the cerebral cortex during a therapeutic course and has positive effects on functional performance and balance of the lower extremities in healthy adults.^42^

Vestibular dysfunctions in the elderly may lead to various psychological problems, including anxiety, depression, or emotional distress. Before initiating a VRT program, the majority of our patients showed BAI scores corresponding to a low degree of anxiety. Our findings exhibited a significantly reduced score for the post-treatment BAI values compared to the baseline values in both study groups. Our findings demonstrate that VRT is effective in treating vestibular disorders in individuals with symptoms of psychological distress such as anxiety. Several studies have reported that tDCS improves cognitive control over negative stimuli such as depression and symptoms of psychiatric disorders such as anxiety. It is hypothesized that bifrontal tDCS over DLPFC is able to modulate dizziness symptoms through strengthening cognitive control over frontolimbic neural networks.^43^, ^44^ However, no significant between-group differences were observed with respect to BAI data across different time points.

The findings of this study also exhibited that multisession tDCS is a safe treatment option for chronic vestibular deficits. The repeated sessions of tDCS were well tolerated by all of the patients in the study and none of the patients reported skin irritation or other adverse effects to stop examination.

## Conclusion

These findings suggest that the application of tDCS over DLPFC could be a practical neuromodulation approach to reduce vestibular symptoms in the elderly patients. Our findings indicated that vestibular rehabilitation therapy, in combination with tDCS, resulted in rapid improvement in dizziness-related disability and balance confidence in individuals with a chronic vestibular disorder.

## Conflicts of interest

The authors declare no conflicts of interest.
